# Human Trophoblast Cells Modulate Endometrial Cells Nuclear Factor κB Response to Flagellin *In Vitro*

**DOI:** 10.1371/journal.pone.0039441

**Published:** 2013-01-08

**Authors:** Ignacio Caballero, Sumiah Al Ghareeb, Shaghayegh Basatvat, Javier A. Sánchez-López, Mehrnaz Montazeri, Nasim Maslehat, Sarah Elliott, Neil R. Chapman, Alireza Fazeli

**Affiliations:** Academic Unit of Reproductive and Developmental Medicine, Department of Human Metabolism, The Medical School, University of Sheffield, Sheffield, United Kingdom; Otto-von-Guericke University Magdeburg, Germany

## Abstract

**Background:**

Implantation is a complex process that requires a delicate cooperation between the immune and reproductive system. Any interference in the fine balance could result in embryo loss and infertility. We have recently shown that Toll-like receptor 5 activation results in a decrease of trophoblast cells binding to endometrial cells in an *in vitro* model of human implantation. However, little is known about the downstream signalling leading to the observed failure in implantation and the factors that modulate this immune response.

**Methods and Principal Findings:**

An *in vitro* model of embryo implantation was used to evaluate the effect of trophoblasts and flagellin on the activation of NF-κB in endometrial cells and whether TLR5-related *in vitro* implantation failure is signalled through NF-κB. We generated two different NF-κB reporting cell lines by transfecting either an immortalized endometrial epithelial cell line (hTERT-EECs) or a human endometrial carcinoma cell line (Ishikawa 3-H-12) with a plasmid containing the secreted alkaline phosphatase (SEAP) under the control of five NF-κB sites. The presence of trophoblast cells as well as flagellin increased NF-κB activity when compared to controls. The NF-κB activation induced by flagellin was further increased by the addition of trophoblast cells. Moreover, blocking NF-κB signalling with a specific inhibitor (BAY11-7082) was able to restore the binding ability of our trophoblast cell line to the endometrial monolayer.

**Conclusions:**

These are the first results showing a local effect of the trophoblasts on the innate immune response of the endometrial epithelium. Moreover, we show that implantation failure caused by intrauterine infections could be associated with abnormal levels of NF-κB activation. Further studies are needed to evaluate the target genes through which NF-κB activation after TLR5 stimulation lead to failure in implantation and the effect of the embryo on those genes. Understanding these pathways could help in the diagnosis and treatment of implantation failure cases.

## Introduction

Implantation of the embryo in the uterus is considered to be one of the most critical steps during pregnancy. This complex biological process represents a paradoxical immune status where a semi-allogenic body (embryo), which under normal circumstances would be rejected by the recipient immune system, is nourished and nurtured [1]. In this regard, different microarray studies have shown that a tight control of the maternal immune system is necessary to promote immune tolerance to the conceptus whilst protecting against infection during the implantation period [1], [2]. However, the mechanisms through which all these processes are regulated are unclear. A successful implantation is dependent on a two-way crosstalk between the embryo and maternal signals [3]. This embryo-maternal dialogue should provide endometrial receptivity in synchrony with an optimal embryo development [4].

Providing appropriate endometrial receptivity is crucial for implantation since approximately two-thirds of implantation failures are imputable to inadequate uterine receptivity [5]. Uterine receptivity to the embryo is clearly influenced by the hormones, growth factors and cytokines present in the uterine environment during the window of implantation. This cytokine network is extremely sensitive to systemic and local changes and needs to be kept in balance for a successful implantation [6], [7]. One of the main regulators of the immune response is the Toll-like receptor family (TLR). TLRs are the main family of pattern recognition receptors (PRRs) of the innate immune system [8], [9]. This family of receptors have been seen to be expressed in human endometrial tissue and trophoblasts [10], [11] and are known to have a key role in the modulation of immune and inflammatory responses in mammals [12]. Although their principal role has been generally assumed to be the defence against infection, TLRs are able to modulate the cytokine environment in response to endogenous factors called “danger-associated molecular patterns” (DAMPs) [13], [14].

TLR signalling involves activation of nuclear factor κB transcription factor (NF-κB). There are two best-described pathways, the canonical and non-canonical, leading to NF-κB activation. In the canonical pathway NF-κB proteins are bound to IκBα in the cytoplasm, preventing its translocation to the nucleus. Upon stimulation, IκBα will be phosphorylated and degraded, allowing the NF-κB dimers to move into the nucleus and bind to the DNA, which will trigger the expression of genes involved in a great array of inflammatory processes [15]. Many of the genes whose expression is influenced by the NF-κB system, such as cyclooxygenase-II (COX2), leukemia inhibitory factor (LIF), colony-stimulating factor-1, are closely related to implantation, suggesting that NF-κB could be a key factor in the regulation of different events at the time of implantation [16]. Recent studies have supported the importance of the TLR family during embryo implantation. It has been reported that activation of TLR5 on endometrial cells by its agonist flagellin resulted in a decrease in number of trophoblast cells binding to endometrial cells in an *in vitro* model of human implantation. These findings suggest that the presence of infection in the uterus is able to disturb the fine balance of cytokines/chemokines in the female reproductive tract (FRT) through the TLR pathway leading to implantation failure [17].

To achieve a complete understanding of the role of the TLR family and the innate immune system in this early stages of pregnancy, it is required to understand how the different factors involved in the embryo-maternal crosstalk modulate the immune response. It is noteworthy that trophoblast cells are potent modulators of the immune response. They are able to secrete different cytokines/chemokines that can influence the response of the maternal immune cells, which would affect their response to infection [11]. In this study, we aim to gain an understanding of the role of the trophoblasts in the modulation of the innate immunity during the maternal interaction with the embryo. Particularly, we evaluated the effect of the trophoblasts and flagellin in the activation of NF-κB in endometrial cells and whether TLR5-related *in vitro* implantation failure is signalled through NF-κB. For this purpose we developed a NF-κB reporting endometrial cell line using a non-cancerous immortalized endometrial epithelial cell line (hTERT-EECs). This method of immortalization brings minimum changes to the cell line and produces immortalized cells that mimic *in vitro*, the *in vivo* molecular and cellular events [18]. Although the hTERT-EECs cell line was established at the proliferative phase, it can bind trophoblast spheroids, which is the main characteristic of a receptive endometrium [17], [18], [19]. Trophoblasts cells were represented by embryo shape spheroids formed from a choriocarcinoma cell line monolayer (JAr cells) [20]. All together, the above mentioned characteristics plus the expression of TLRs1-3 and 5–10 in the hTERT-EECs [21] make them a suitable choice for our study. However, during the development of this manuscript a new report was published showing that the DNA profile of the hTERT-EEC resembled that of MCF-7 epithelial breast cancer cells, and suggested that results obtained using the hTERT-EEC should be interpreted with caution [22]. Due to this claim, we decided to confirm our results using another endometrial cell line, in this case the Ishikawa 3-H-12 cell line, which corroborated the results obtained using hTERT-EEC.

We observed that both the presence of trophoblasts and flagellin increased NF-κB activity in both endometrial cell lines. Moreover, TLR5 response in both cell lines to flagellin was enhanced in the presence of trophoblast cells. Finally, application of a NF-κB inhibitor was able to restore the attachment of JAr spheroids to the endometrial cell monolayer in both cell lines. These results suggest that embryo arrival to the uterus have a regulatory effect on the modulation of innate immune activity in the female reproductive tract and flagellin inhibitory effect on trophoblast cells attachment to endometrial cells is mediated through activation of NF-κB pathway in these cells.

## Methods

### Cell lines and culture

An *in vitro* model of human implantation that had been previously established in our laboratory was used to mimic the *in vivo* molecular and cellular changes that occur during implantation [17]. The telomerase immortalized endometrial epithelial cell line (hTERT-EEC) [18] was kindly gifted by Dr. Sabine Hombach-Klonisch (University of Manitoba, Winnipeg, MB, Canada). The human endometrial adenocarcinoma Ishikawa cell line [21] was a kind gift of Dr. S. M. Laird (Sheffield Hallam University, UK). Both these cell lines were representing a receptive endometrium. A human choriocarcinoma cell line (JAr) from first trimester trophoblasts, acquired from ATCC (Cat. No. HTB-144) was used to mimic trophoblast cells *in vitro*. The hTERT-EECs were cultured at 37°C in an atmosphere with 5% CO_2_ until they reached confluence in T75 flasks and then transferred gently to 12-well plates in DMEM-F12 (Sigma, Cat. No. D8437, Irvine, UK) supplemented with 1% Penicillin and Streptomycin (P/S Sigma, Cat. No. PO781-100ML, St. Louis, MO), 10% Fetal Bovine Serum (FBS) (Sigma, Cat. No. F 9665, Irvine, UK), 1% l-glutamine (Sigma, Cat. No. G7513, Irvine, the UK) and 160 ng/ml Insulin (Human recombinant insulin, Gibco Invitrogen, Cat. No. 12585-014, Denmark). Ishikawa cells were cultured at 37°C in an atmosphere with 5% CO_2_ in T75 flasks and then transferred gently to 12-well plates in DMEM-F12 (Sigma, Cat. No. D8437, Irvine, UK) supplemented with 1% Penicillin and Streptomycin (P/S Sigma, Cat. No. PO781-100ML, St. Louis, MO), 10% Fetal Bovine Serum (FBS) (Sigma, Cat. No. F 9665, Irvine, UK), 1% l-glutamine (Sigma, Cat. No. G7513, Irvine, the UK). JAr cells were cultured in a T75 flask in RPMI 1640 media (Sigma, Cat. No. R0883, Irvine, the UK) supplemented with 10% FBS (Sigma), 1% l-glutamine (Sigma) and 1% P/S (Sigma). JAr cells were grown at 37°C in 5% CO_2_ atmosphere until confluent. In both cell types, the media was changed every second day. At confluence, hTERT-EECs and JAr cells were washed using Ca^2+^ and Mg^2+^ free Dulbecco's phosphate-buffered saline (DPBS Sigma, Cat.No. D1408, Irvine, UK). The cells were then harvested using trypsin-EDTA (Sigma, Cat. No. T3924, Irvine, UK) and then pelleted by centrifugation at 300 g for 4 minutes.

### Generation of JAr spheroids

To form multicellular spheroids with a diameter of 200–250 µm from JAr cells monolayer [19], [23], [24], 1×10^6^ cells/ml were counted with a haemocytometer and then cultured in 5 ml of supplemented RPMI 1640 medium (Sigma) in 60×15 mm Petri dishes (CellStar tissue culture dishes, Greiner Bio-One, GmbH/Germany) at 37°C in 5% CO_2_. They were kept on a gyratory shaker set at 250 rpm for 24 h. The resulting spheres were gently transferred into a 35×10 mm Suspension Petri dish (Corning Incorporated, Corning, NY14831 USA) containing 1 ml of RPMI 1640 supplemented media [17].

### Nuclear extract preparation

Nuclear extracts from hTERT-EECs were prepared as detailed in [25]. Briefly, cells were harvested by centrifugation at 1000 rpm for 5 min and washed with nuclear extraction buffer A (NEBA; 10 mM HEPES, pH 7.9, 1.5 mM MgCl_2_, 10 mM KCl, 1 mM dithiothreitol and protease inhibitors). The cell pellets were then resuspended in NEBA containing 0.1% of Igepal CA-630 (Sigma) and incubated on ice for 5 min. The cell lysate was centrifuged at 14000 rpm for 15 min at 4°C and the pellet resuspended in high salt buffer (25% glycerol 20 mM HEPES, pH 7.9, 1.5 mM MgCl_2_, 420 mM NaCl, 1 mM dithiothreitol and protease inhibitors) and incubated on ice for 30 min. The suspension was centrifuged at 14000 rpm for 15 min at 4°C and the supernatant (nuclear extract) was aliquoted, snap frozen in liquid nitrogen and stored at −80°C.

### Determination of NF-κB activity

NF-κB activity was determined using either electrophoretic mobility shift assays (EMSA) or a NF-κB reporting endometrial cell line.

EMSA was performed using a radiolabelled ([α-^32^P]dATP) NF- κB consensus oligonucleotide (5′-GATCCGCTG**GGGACTTTCC**AGGC G-3′; κB site in bold). Five µg of crude nuclear extract was incubated in a 20 µl reaction volume consisting of 20 mM HEPES (pH 7.9), 1 µg poly (dI-dC∶dI-dC) and approximately 0.1 ng α-^32^P-labeled double stranded oligonucleotide. To assess the specificity of binding, 100 ng (1000-fold excess) of the respective cold (unlabelled) specific κB or nonspecific (5′-GATCCACTCAGAC**CACGTG**GTCGGGTAC-3′; c-Myc binding site in bold) oligonucleotides were included with the labelled probed in each reaction. Samples were incubated for 15 min and then DNA∶protein complexes were resolved using 1× Tris/glycine/EDTA [25 mM Tris (pH 8.0), 190 mM glycine, 1 mM EDTA] and 4% non-denaturing polyacrylamide gels.

Evaluation of NF-κB activity using the endometrial reporter cell line was as follows. The endometrial cell lines, either hTERT-EECs or Ishikawa, were seeded and grown in a 12-well plate until 70% confluency and transiently transfected with pNifty2-SEAP (Invivogen) using X-tremeGENE HP DNA transfection reagent (Roche) following manufacturer's instructions. For each experiment, 25 µl of the supernatant from the transfected endometrial cells stimulated as below described was collected. SEAP in the supernatant was detected using either QUANTI-Blue™ (Invivogen) or NovaBright™ Secreted Placental Alkaline Phosphatase (SEAP) Enzyme Reporter Gene Chemiluminescent Detection System 2.0 kit (Invitrogen) following the manufacturer's protocol. Samples assayed using the QUANTI-Blue™ revealed samples were quantified as OD at 620 nm using a microplate reader (Multiskan). When higher sensitivity was required in the assay, Novabright™ kit was employed. The results from this chemiluminescent assay were read using a Sirius Luminometer (Berthold detection systems; Geneflow Staffs. UK). Data from all experiments are reported as the fold induction of SEAP activity over untreated controls.

### Effect of JAr cells co-incubation with endometrial cells on NF-κB activation and attachment to the endometrial cell monolayer

To find out the effect of JAr cells on NF-κB activation, both endometrial cell lines were grown in triplicate to 60% confluence. They were then transfected with pNifty2-SEAP Plasmid as described above. After 24 hours of incubation at 37°C, different number of JAr spheroids (0, 20, 50 or 100) were gently delivered to each well of the 12 well plates. Samples were collected at 0, 2, 6 and 24 hours and analysed using NovaBright™ Secreted Placental Alkaline Phosphatase (SEAP) Enzyme Reporter Gene Chemiluminescent Detection System 2.0. To evaluate whether the number of JAr spheroids added to the co-culture influence their attachment to the endometrial cell monolayer, JAr spheroids were gently added to the hTERT-EEC monolayer and co-incubated for 1 or 24 hours. The plates were then washed twice on a horizontal shaking device for 4 min, at 200 rpm and the media discarded by inverting the plate. The tightly attached spheroids were counted and the total percentage of the remaining spheroids was calculated.

### Effect of flagellin treatment on NF-κB activation

To find out the effect of flagellin (Invivogen) on NF-κB activation, both endometrial cell lines were grown in triplicate to 60% confluence before transfection with the pNifty2-SEAP plasmid. Transfected cells were stimulated with different concentrations of flagellin (0, 10, 100, 500 ng/ml) for 24 hours. Samples were analysed at 0, 2, 4 and 24 hours using QUANTI-Blue™. EMSA was performed on hTERT-EECs either incubated or not with 100 ng/ml of flagellin for 24 h to confirm the results observed using the reporter cell line. Cell viability was evaluated using ethidium homodimer/Calcein-AM following manufacturer's instructions (Viability/Cytotoxicity kit, Molecular Probes, Eugene, Oregon, USA).

### Effect of JAr cells on the endometrial response to flagellin

To investigate whether the trophoblast cells are able to modulate TLR5 response to flagellin a 2×2 factorial experiment was designed. The factors included in the experiment were as follows: flagellin concentration (0, 100 ng/ml) and the presence or absence of JAr spheres. Briefly, Both endometrial cell lines were cultured in triplicate in 12 well plates until 60% confluent and transfected with pNifty2-SEAP plasmid as previously described. Twenty-four hours after transfection, 100 JAr spheres were gently delivered to the endometrial monolayer and co-incubated for 1 h before the addition or omission of 100 ng/ml of flagellin. Supernatants were collected at 0, 2, 6 and 24 hours after addition of flagellin and analysed using the NovaBright™ Secreted Placental Alkaline Phosphatase (SEAP) Enzyme Reporter Gene Chemiluminescent Detection System 2.0.

### Effect of different concentration of glass beads on NF-κB activation

This experiment was performed to determine whether the observed effect of JAr cells in NF-κB activity was related to the mere physical contact of a foreign body with the endometrial monolayer or to specific signalling from the JAr cells. Briefly, hTERT-EECs were transfected with pNifty2-SEAP plasmid as described above. Transfected hTERT-EECs were co-incubated with either 0, 1 or 5 µl of a solution of glass beads (1×10^5^ beads/ml PBS buffer; Sigma, Cat. No. G-8893, Steinheim, Germaney), representing 0, 100 and 500 beads, respectively. After 24 h of co-incubation the samples were collected and analysed using the NovaBright™ Secreted Placental Alkaline Phosphatase (SEAP) Enzyme Reporter Gene Chemiluminescent Detection System 2.0.

Similarly, we evaluated the effect of glass beads on TLR5 response to flagellin. Transfected hTERT-EECs were co-incubated with 1 µl of the glass beads solution (100 beads) for 1 h. Thereafter, the hTERT-EECs-glass beads co-culture was treated with 100 ng/ml of flagellin for 24 h before sample collection. Samples were then analysed using the NovaBright™ Secreted Placental Alkaline Phosphatase (SEAP) Enzyme Reporter Gene Chemiluminescent Detection System 2.0.

### Effect of blocking NF-κB on JAr spheroids attachment to the endometrial cells in the presence of flagellin

To find out if there is any relationship between NF-κB activation and *in vitro* implantation failure caused by the presence of flagellin [17], Both endometrial cell lines were transfected with pNifty2-SEAP as previously described. After 24 hours, either 10 µM BAY11-7082, a NF-κB inhibitor (Invivogen, Cat. No. Tlrl-b82, San Diego, CA, USA) or the equivalent quantity of vehicle (1 µl of DMSO) was added to the endometrial cells and incubated for 30 minutes. Thereafter, hTERT-EECs were either stimulated or not with 10 ng/ml of flagellin (Invivogen) and incubated for 6 hours while the Ishikawa cells were either stimulated or not with 100 ng/ml of flagellin (Invivogen) and incubated for 24 hours. After incubation with flagellin, 50 JAr spheroids were gently added to each well and co-incubated for one hour. The plates were then washed twice as above described and the total percentage of the remaining spheroids was calculated. NF-κB activation was measured in the hTERT-EECs at 6 h after the addition of flagellin using the NovaBright™ Secreted Placental Alkaline Phosphatase (SEAP) Enzyme Reporter Gene Chemiluminescent Detection System 2.0.

### Statistical analysis

The results were expressed as mean ± SEM of at least 3 different replicates. Data of NF-κB activity are reported as the fold induction of SEAP activity over untreated controls. Statistical analysis was performed using ANOVA (SPSS version 19.0; SPSS inc, Chicago, IL, USA). When ANOVA revealed a significant effect, values were compared using the Bonferroni test and were considered significant at p<0.05.

## Results

### Effect of JAr cells co-incubation with endometrial cells on NF-κB activation and attachment to the endometrial cell monolayer

**Figure 1:**
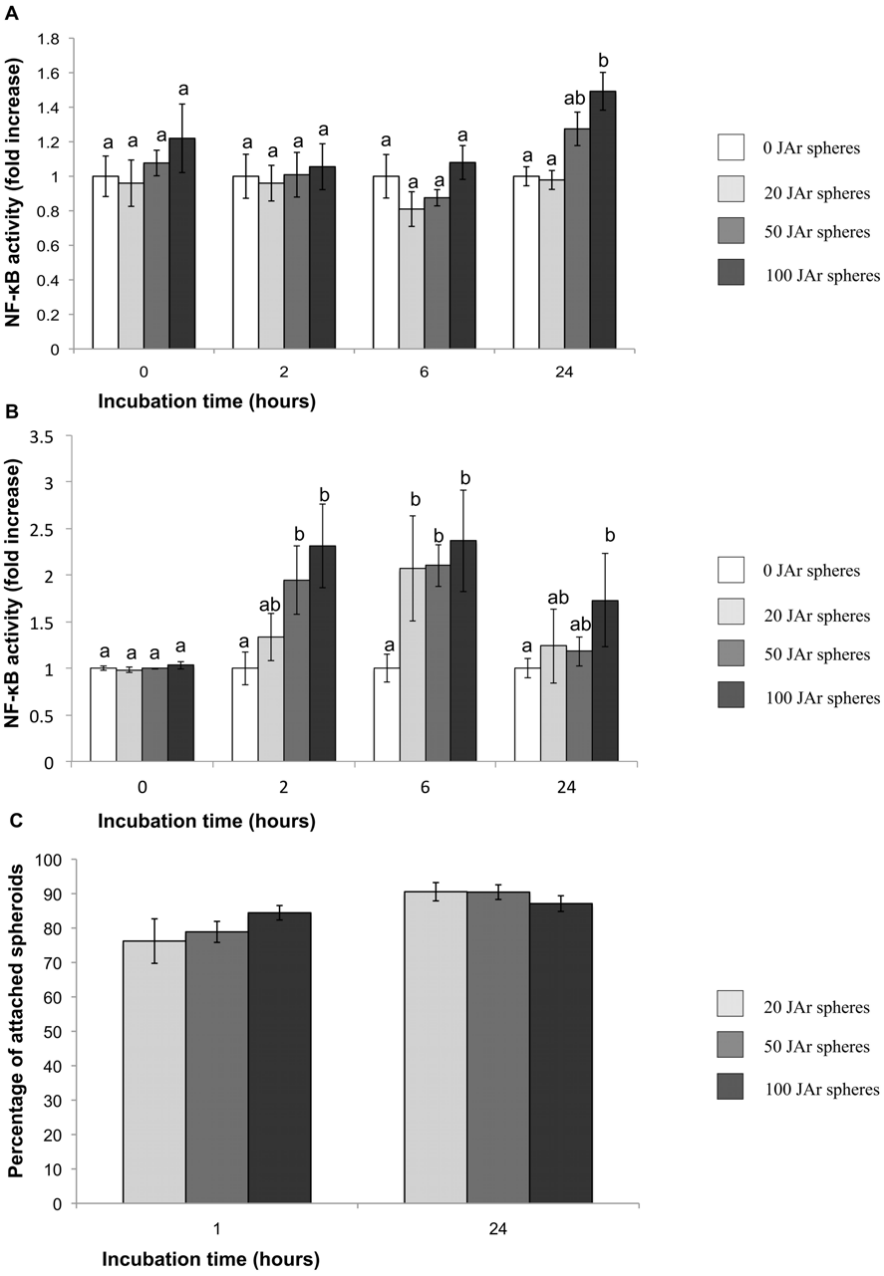
Effect of different concentrations of JAr spheres on hTERT-EECs NF-κB activity and attachment to the endometrial cell monolayer. (A) hTERT-EECs and (B) Ishikawa 3-H-12 cells transfected with the SEAP reporter gene were co-cultured with 0, 20, 50 or 100 JAr spheroids for 24 hours. Samples were collected at 0, 2, 6 and 24 hours and analyzed using NovaBright™ Secreted Placental Alkaline Phosphatase (SEAP) Enzyme Reporter Gene Chemiluminescent Detection System 2.0. Data of NF-κB activity are reported as the fold induction of SEAP activity over untreated controls. Different letters mean significant difference (p<0.05). (C) hTERT-EECs were co-cultured with 20, 50 or 100 JAr spheroids for either 1 or 24 hours. The plates were then washed and the percentage of the attached spheroids was calculated.

No differences were found when JAr spheroids were added to the hTERT-EECs during the first 6 h of co-incubation regardless of the number of spheres added (p>0.05). However, after 24 h of co-incubation time, the addition of 50 or more JAr spheres to the endometrial monolayer increased the levels of NF-κB activation when compared to controls (p<0.05; Fig. 1A). On the other hand, the incubation of JAr spheroids with the Ishikawa cells showed a significant increase in NF-κB activation already at 2 h of co-incubation time (p<0.05: Fig. 1B). No significant changes in the percentage of JAr spheroids attached to the hTERT-EEC endometrial monolayer were observed regardless the number of JAr spheres added (Fig. 1C).

### Effect of flagellin treatment in NF-κB activation

**Figure 2:**
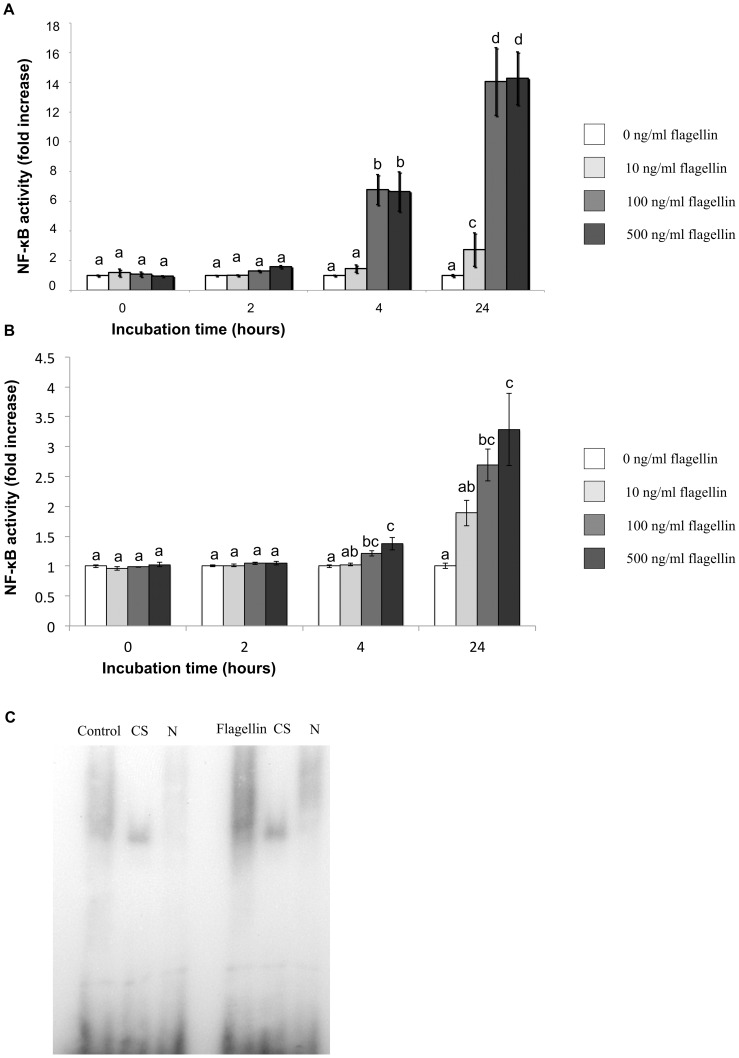
Effect of flagellin treatment on hTERT-EECs NF-κB activity. (A) hTERT-EECs and (B) Ishikawa 3-H-12 cells transfected with the SEAP reporter gene were co-cultured with 0, 10, 100 or 500 ng/ml of flagellin for 24 hours. Samples were collected at 0, 2, 4 and 24 hours and analyzed using Quantiblue™. Data of NF-κB activity are reported as the fold induction of SEAP activity over untreated controls. Different letters mean significant difference (p<0.05). (C) Electrophoretic mobility shift assays (EMSA) analysis of DNA-binding activity of NF-κB in hTERT-EECs treated with (flagellin group) or without (control group) 100 ng/ml of flagellin for 24 hours. Control reactions were incubated with either excess (1000 fold) unlabeled κB (cold specific; CS) or nonspecific cold probe (N) before reaction with labeled NF-κB probe.

Treatment of hTERT-EECs and Ishikawa with different concentrations of flagellin significantly increased NF-κB activity in a concentration-dependent manner (p<0.05). However, no further increase was observed with flagellin concentrations above 100 ng/ml. This increase was time-dependent with higher doses of flagellin (100 and 500 ng/ml) showing a significant effect already at 4 hours while the lowest dose of flagellin needed 24 hours to show any significant effect (Fig. 2A and 2B). NF-κB activation by flagellin was confirmed by EMSA, where an increased DNA-binding activity to κB consensus sequence was observed in those samples treated with 100 ng of flagellin when compared to controls (Fig. 2C).

### Effect of JAr spheres on the endometrial cells response to flagellin

**Figure 3:**
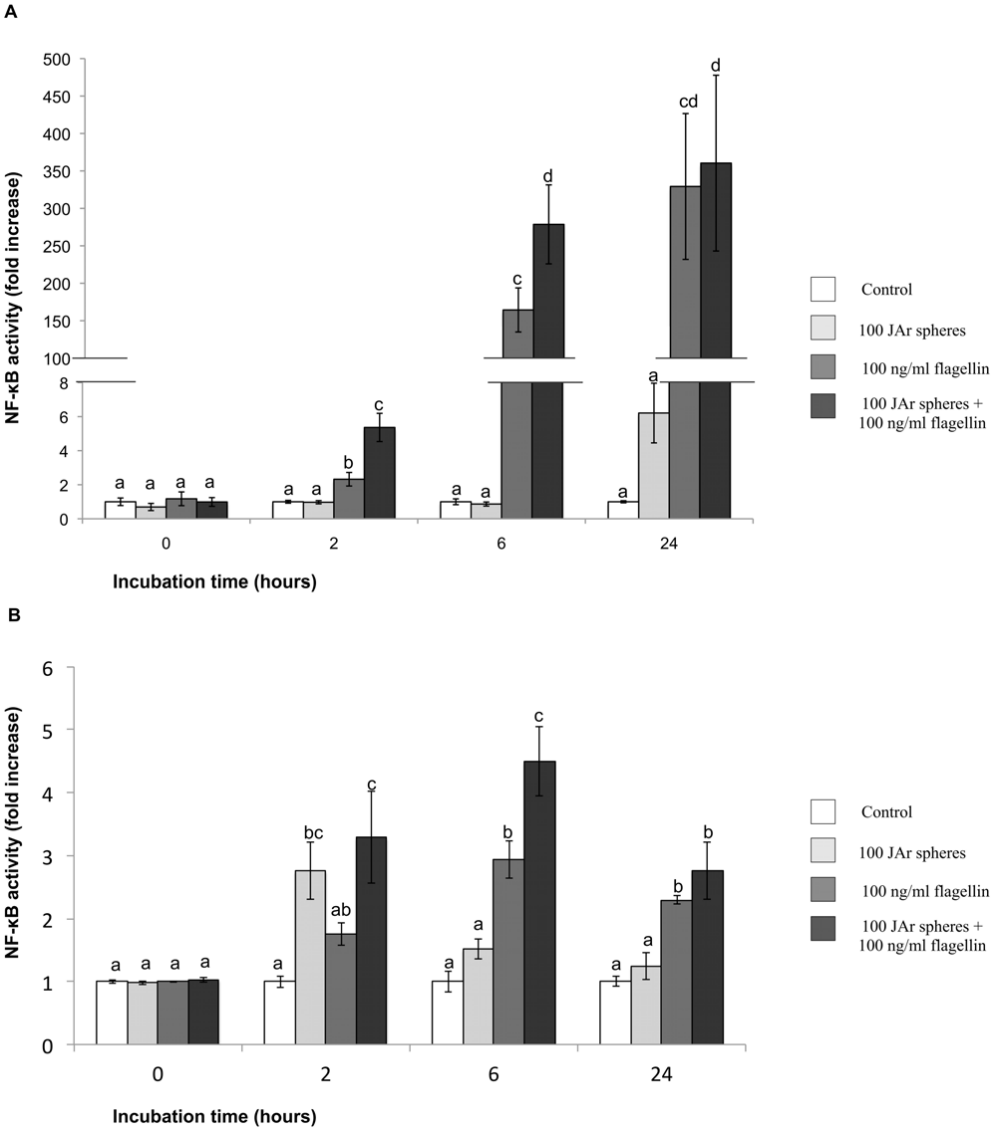
Effect of JAr spheres on the endometrial response to flagellin. A 2×2 factorial experimental design was developed were (A) hTERT-EECs or (B) Ishikawa 3-H-12 cells transfected with the SEAP reporter were either: cultured in the absence of both JAr spheres and flagellin (control); cultured with 100 JAr spheres; cultured with 100 ng/ml of flagellin or pre-incubated with 100 JAr spheres for 1 h before the addition of 100 ng/ml of flagellin. Samples were collected at 0, 2, 6 and 24 hours and analyzed using NovaBright™ Secreted Placental Alkaline Phosphatase (SEAP) Enzyme Reporter Gene Chemiluminescent Detection System 2.0. Control Data of NF-κB activity are reported as the fold induction of SEAP activity over untreated controls. Different letters mean significant difference (p<0.05).

As expected, treatment of hTERT-EECs with flagellin significantly increased NF-κB activity. The NF-κB activation induced by flagellin was further increased significantly by the addition of JAr spheres after 6 h of co-incubation (p<0.05). However, no differences were observed with incubation times as long as 24 h (p>0.05; Fig. 3A). Similarly, NF-κB activation induced by flagellin in the Ishikawa cells was further increased significantly by the addition of JAr spheres after 2 and 6 h of co-incubation (p<0.05; Fig. 3B). No effect on cell viability or morphology was observed after the addition of 100 ng/ml of flagellin in both endometrial and trophoblast cells (data not shown).

### Effect of different concentration of glass beads on NF-κB activation

**Figure 4:**
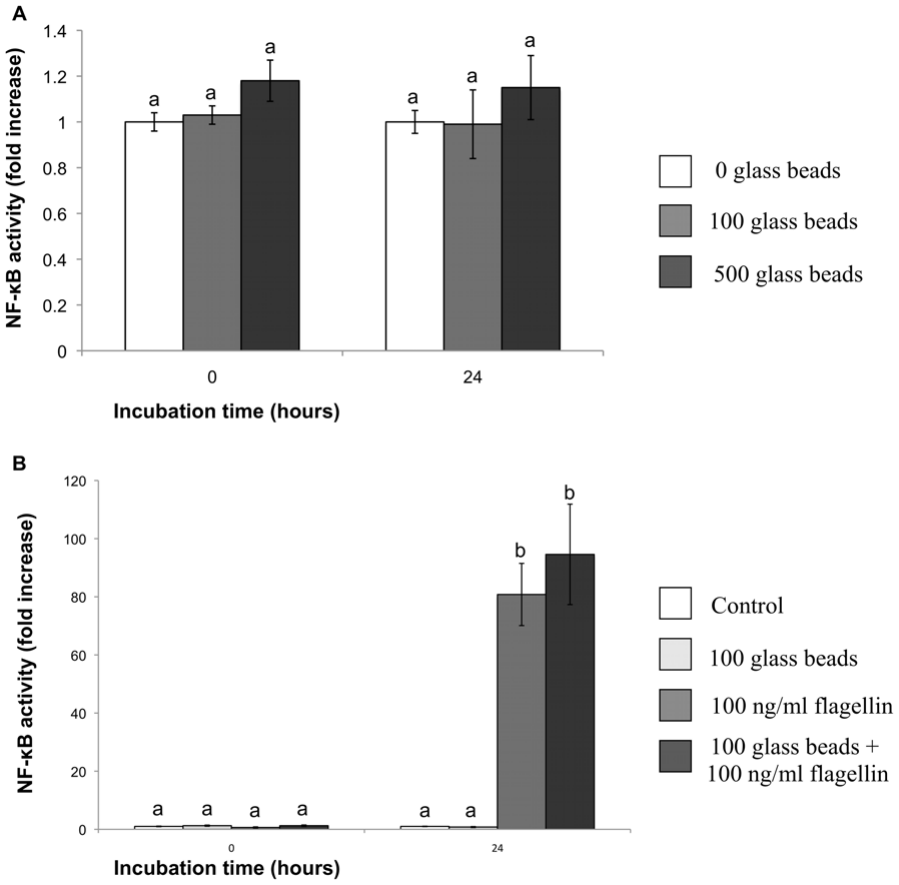
Effect of glass beads on hTERT-EECs NF-κB activity. hTERT-EECs transfected with the SEAP reporter gene were co-cultured either with: (A) 0, 100, or 500 glass beads; or (B) cultured in the absence of both glass beads and flagellin (control), cultured with 100 glass beads, cultured with 100 ng/ml of flagellin, or pre-incubated with 100 glass beads for 1 h before the addition of 100 ng/ml of flagellin. Samples were collected at 0, 24 hours and analyzed using NovaBright™ Secreted Placental Alkaline Phosphatase (SEAP) Enzyme Reporter Gene Chemiluminescent Detection System 2.0. Control Data of NF-κB activity are reported as the fold induction of SEAP activity over untreated controls. Different letters mean significant difference (p<0.05).

Unlike the observed effect of JAr spheres on NF-κB activation in hTERT-EECs, none of the glass beads concentrations (100 and 500 glass beads) added to the hTERT-EECs produced a significant change in NF-κB activity after 24 h of co-incubation (p>0.05; Fig. 4A). In addition, no effect on TLR5 response to flagellin was observed when hTERT-EECs were co-incubated with glass beads for 1 h before the addition of flagellin (p>0.05; Fig. 4B).

### Effect of blocking NF-κB on JAr spheroid attachment to the endometrial cells in the presence of flagellin

**Figure 5:**
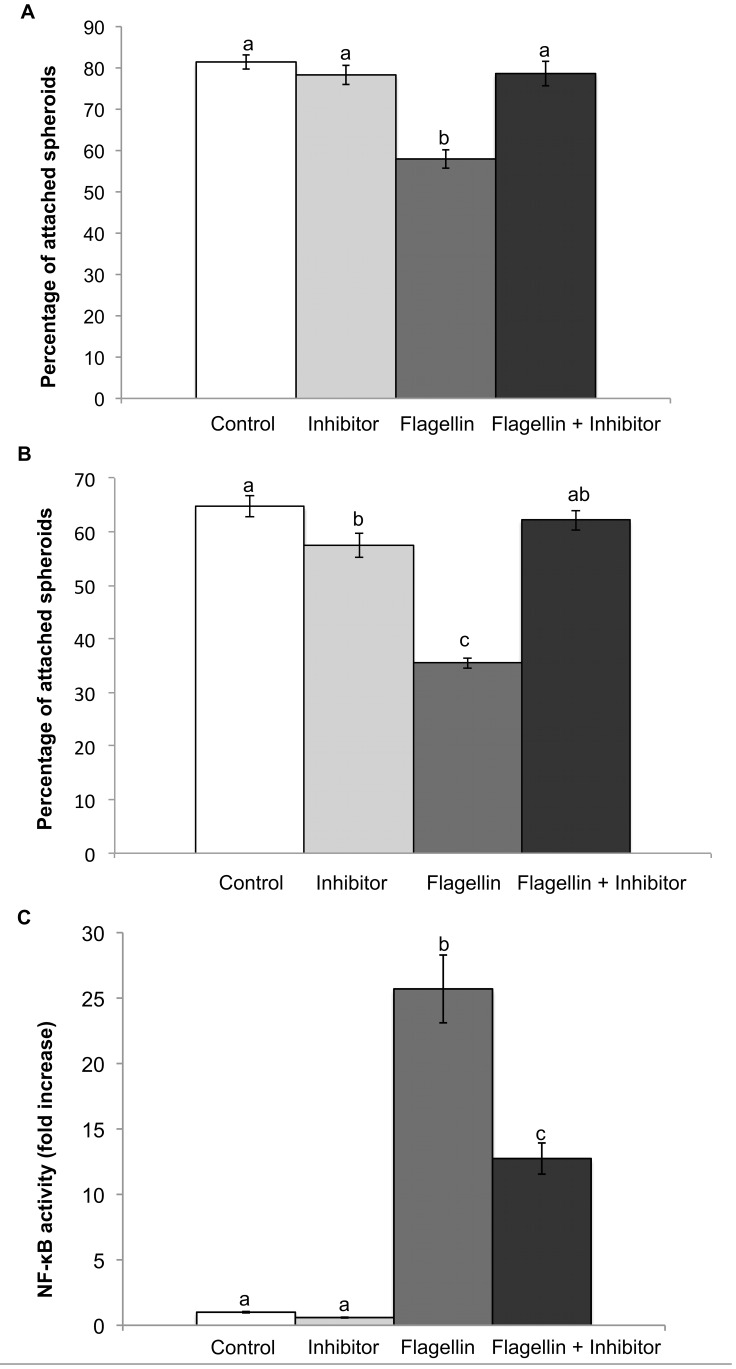
Effect of blocking NF-κB on JAr spheroids attachments to the Endometrial cells in the presence of Flagellin. (A) hTERT-EECs or (B) Ishikawa 3-H-12 cells transfected with the SEAP reporter gene were pre-treated for 30 min with either 10 µM BAY11-7082 (inhibitor) or the equivalent quantity of vehicle (Control; 1 µl of DMSO). Thereafter, hTERT-EECs were either stimulated or not with 10 ng/ml of flagellin and incubated for 6 hours, while the Ishikawa 3-H-12 cells were either stimulated or not with 100 ng/ml of flagellin and incubated for 24 hours. After the incubation time with flagellin, 50 JAr sheroids were gently added to each well and co-incubated for one hour. The plates were then washed and the percentage of the attached spheroids was calculated. (C) NF-κB activation was measured in the hTERT-EEC at 6 h after addition of flagellin using NovaBright™ Secreted Placental Alkaline Phosphatase (SEAP) Enzyme Reporter Gene Chemiluminescent Detection System 2.0. Control Data of NF-κB activity are reported as the fold induction of SEAP activity over untreated controls. Different letters mean significant difference (p<0.05).

Treatment of both endometrial cell lines with flagellin decreased the number of attached JAr spheroids to the endometrial monolayer when compared to non-treated controls (p<0.05). However, a 30 min pre-incubation of the hTERT-EECs with 10 µM of BAY11-7082 before the addition of flagellin significantly restored the percentage of attached JAr spheroids to levels similar to the control (p<0.05) (Fig. 5A and 5B). Moreover, the pre-incubation of hTERT-EECs for 30 min with 10 µM of BAY11-7082 (NF-κB inhibitor) significantly reduced NF-κB activation induced by flagellin (p<0.05; Fig. 5C).

## Discussion

Pregnancy is a complex immunological process, where the inflammatory environment of the uterus shifts from a pro-inflammatory to an anti-inflammatory profile depending on the pregnancy stage. Implantation requires a strong inflammatory response. Several immune cells are found in the uterus at the site of implantation accumulating around the trophoblast cells, which researchers has taken as evidence that the maternal immune system reacts to the invading trophoblasts. Although this reaction could seem to be an immune response against the semi-allogenic fetus, different studies have shown that this inflammatory response is necessary to support the fetal development [11], [26]. However, the mechanisms through which the trophoblast and the maternal immune system collaborate to protect the maternal tract and the fetus against infection instead of rejecting the fetal allograft are not fully known. The results from our experiments clearly showed that local signals from the trophoblast cells are able to modulate the immune response in the endometrium, at least *in vitro*. The addition of trophoblasts significantly increased NF-κB activity in both hTERT-EEC and Ishikawa cells and had a synergistic effect on flagellin-derived NF-κB activation. Moreover, the canonical NF-κB signalling seems to be involved in flagellin-induced implantation failure since blocking its activation with BAY11-7082 (an IκB phosphorylation inhibitor) restored the binding of JAr spheroids to the flagellin-stimulated endometrial monolayer to levels similar to non-stimulated controls.

Increased binding of NF-κB to DNA has been previously reported *in vivo* at the time of implantation in mice [27]. In the same line of evidence, immunohistological studies have shown a translocation of NF-κB from the cytoplasm to the nucleus of the luminal epithelial cells on day 12–13 in pregnant pigs [28]. The importance of this phenomenon is highlighted by the fact that suppression of NF-κB activation produced a delay in the timing of the implantation in mice [16]. Although all the data points toward an increase in NF-κB activity during the implantation, the mechanism through which this activation happens is still unknown. At the time of implantation the endometrium is subjected to systemic and local signals from the mother and the embryo. *In vivo*, it has been reported that steroid hormones and their receptors modulate NF-κB activity [29]–[31]. However, activation of NF-κB in the endometrium appears to be related not only to estrogen or progesterone concentrations. In our study, we employed an *in vitro* system where the endometrium was not subjected to the influence of hormones and, therefore, only the signals from the trophoblast could affect the hTERT-EECs or Ishikawa cells response. We could observe that the addition of 100 JAr spheroids increased the levels of NF-κB activation after 24 h of co-incubation with hTERT-EECs. A similar effect was observed in the Ishikawa cells already after 2 h of co-incubation. In addition, the presence of JAr spheroids was also able to increase NF-κB activity in the hTERT-EECs and Ishikawa cells subjected to flagellin treatment for 6 hours. This data together with the fact that no effect in NF-κB activity was observed by replacing JAr spheroids with glass beads strongly suggest that molecular signals from the trophoblasts have a direct effect in the endometrium immune response.

Trophoblasts cells are thought to be important modulators of the immune response. They are known to secrete different factors that can activate NF-κB, such as LIF, Tumour necrosis factor-α and Interleukin-1β (IL-1β) [16], [32]. The IL-1 system is a key regulator in many inflammatory processes as well as in the establishment of implantation. They are formed by IL-1α and IL-1β, two IL-1 receptors, IL-1receptor I (IL-1RI), and II (IL-1RII), which is non-functional and serve as a decoy, and the IL-1 receptor antagonist (IL-1ra). Both IL-1α and IL-1β are able to bind to receptor IL-1RI starting a signalling cascade that will lead to NF-κB activation [33]. High levels of IL-1β have been found to be secreted by cytotrophoblasts from first trimester placenta in human and pig trophoblasts at the time of implantation [34], [35]. IL-1β have been seen to be expressed in the JAr cells [36], and so it seems reasonable that IL-1β released by JAr spheroids could induce the observed NF-κB activation in our endometrial cell line. This point needs to be further investigated in future experiments.

Other mechanisms have been suggested that could lead to an increase in NF-κB activity in the endometrium involving PRRs, more likely the TLR family. TLRs are recently gaining more importance not only as the first mechanism of defence in the innate immune system against infection but also as regulators of the cytokine network involved in the early stages of pregnancy [28]. They are widely expressed at the maternal-fetal interface and have been described in the trophoblasts, the immune cells from the uterine bed and the endometrial epithelium and stroma [10], [11]. Extensive work in the last decade has shown that TLRs could be activated not only by their specific pathogen-associated molecular pattern (PAMPs) but also by endogenous ligands, such as Heat-shock proteins, fibronectin, fibrinogen, surfactant protein-A, heparan sulphate, oligosaccharide of hyaluronan among others [37]. Ross *et al.* [28] found a temporal affiliation between TLR4, receptor activator of NF-κB (TNFRSF11A) and NF-κB inducible genes in the pig uterus suggesting that TLR4 and/or TNFRSF11A could be involved in the activation of NF-κB during the initiation of uterine receptivity. These authors hypothesized that conceptus expression of TLR4 endogenous ligands, fibrinogen and fibronectin, which are up-regulated at the time of implantation, could activate endometrial TLR4 and subsequently trigger the downstream signalling that leads to NF-κB activation [28]. Besides this direct effect on TLR4, these molecules have been shown the capacity to serve as PAMP-binding molecules (PBMs) or PAMP-sensitizing molecules (PSMs). Type III repeat domains of fibronectin present an architecture that suggest they are adapted to promote LPS or presentation to TLR4. Another interesting feature of both fibronectin and fibrinogen is that they are able to enhance the sensitivity of cells toward greater responsiveness to LPS, lipopeptide and CpG, implying a role of these molecules in the modulation of TLR response to their ligands [38].

So far, we have observed that trophoblast cells produced an increase in NF-κB activation in the endometrium and are also able to modulate endometrial response to flagellin either directly or indirectly. However, what could be the biological significance of this effect? Bacterial and viral infections are a threat to pregnancy and fetus well being. TLRs are gatekeepers of the innate immunity, they are able to defend the uterus against infection, but this inflammatory reaction could alter pregnancy outcome. For instance, intrauterine injection of heat-killed *E. coli* into wild type pregnant mice induces pre-term labour whereas no effect is observed in TLR4-mutated mice [11]. On the same line, cervicovaginal TLR4 stimulation with LPS induced implantation failure in mice [39]. Also, stimulation of TLR3 with poly I∶C in mice during pregnancy increased the fetal losses and induced pre-term labour in wild type mice [11], [40]. The mechanisms of action through which TLR activation lead to these detrimental effects are not clear. After binding with their ligands, TLRs activate NF-κB through MyD88 and TRIF-dependent pathway [41], which suggest a possible role of NF-κB signalling in pregnancy failure. In this regard, previous results from our laboratory showed that TLR5 activation in hTERT-EECS results in implantation failure *in vitro* [17]. Here, we observed that pre-treatment of hTERT-EEC or Ishikawa cells with flagellin significantly increased NF-κB activity in a dose-dependent manner but no further effect was observed increasing flagellin concentrations above 100 ng/ml. This is in agreement with results from Aboussahoud *et al.* [21] where 100 ng/ml of bacterial flagellin was the most effective dose that induced IL-8 production in hTERT-EECs, showing no further increases when higher concentrations of flagellin were used. Furthermore, blocking NF-κB activation with a specific inhibitor, we were able to reduce the levels of NF-κB activation induced by flagellin and restore the attachment of JAr spheroids to the endometrial cells in an *in vitro* implantation assay. These evidences clearly show that NF-κB signalling is involved in the suppression of JAr spheroids attachment to the endometrial cell monolayer after TLR5 activation.

As we have mentioned before, NF-κB is a key transcription factor that regulates the expression of a great number of inflammatory genes, influencing cell response to pathogens and stress [41]. One of the genes which transcription is promoted by NF-κB is the IL-1ra gene [42]. Interestingly, TLR5 activation induced the secretion of IL-1ra in intestinal epithelia and macrophages [43]. IL-1ra is known to block the binding of both IL-1α and IL-1β to its receptor, blocking further signal transduction. Mice injected with IL-1ra during the periimplantation period showed impaired embryonic adhesion to the endometrial epithelium, presumably related to the alteration of α4, αv and β3 adhesion molecules [44]. Although, this seems a plausible mechanism to explain how TLR5 activation with flagellin decreases JAr spheroids attachment to the hTERT-EECs and the Ishikawa cells through the NF-κB signalling, further investigations are needed to demonstrate this relationship. However, the following question arises, if the trophoblast cells increase the activity of NF-κB in the endometrium in the presence of an infection, are the same cells that promote fetal acceptance initiating signals that reject the embryo? Our data showed that much higher levels of NF-κB activation are achieved after flagellin stimulation compared to trophoblast. Moreover, NF-κB activation induced by the trophoblast cells did not affect the percentage of JAr spheroids attached to the endometrial monolayer. Then, it seems that a stringent regulation of NF-κB activation is necessary to prevent a severe inflammatory response that could potentially compromise the pregnancy. Besides, it is important to take into account that although nuclear translocation is critical for NF-κB activation, the set of genes for which transcription is promoted by NF-κB is dependent on additional layers of regulation. For example, activation of some target genes may have different requirements and depend on factors such as, cooperative DNA binding with other transcription factors, interaction with coactivators such as CBP/p300 or nuclear IκB proteins, and chromatin structure of the target gene [45]. Although much remains to be learned about these mechanisms, it can be speculated that different physiological settings and stimulus would regulate different set of genes.

During the preparation of this manuscript a report was published pointing to a potential contamination of the hTERT-EEC cells with MCF-7 cells [22]. It has been impossible for us to verify or disregard this claim about the potential contamination of hTERT-EEC cells used in our experiments with MCF7 cells. However, we opted to repeat all the main experiments reported in this manuscript using another endometrial cell line, Ishikawa 3-H-12, to confirm the results obtained with the hTERT-EEC cells. Although there were differences in the sensitivity of these cell lines to TLR ligands, which can be related to differences between cell lines, both cell lines displayed the same type of response to both flagellin and trophoblast cells, confirming the findings reported in this manuscript.

In conclusion, these results suggest that TLR5 response to flagellin in endometrial cells is enhanced in the presence of the trophoblast cells i.e. embryo arrival to the uterus may influence innate immune mediation in the female reproductive tract. Implantation failure caused by intrauterine infections could be associated with abnormal levels of NF-κB activation. Further studies are needed to evaluate the mechanisms through which NF-κB activation after TLR5 stimulation lead to failure in implantation. Understanding these pathways could help in diagnosis of infertility in women and treatment of implantation failure cases.
